# Non-Hodgkin’s lymphoma Breast in a lactating mother : Case Report

**DOI:** 10.3126/nje.v12i1.42975

**Published:** 2022-03-31

**Authors:** Neeraj Kumar Rathee, Nidhi Gupta, Sawant Sharma, Hari Krishan Rathee

**Affiliations:** 1,2,3 Department of Radiation Oncology, Government Medical College and Hospital, Sector-32, Chandigarh, India; 4 Department of General Surgery, All India Institute of Medical Sciences, New Delhi, India

**Keywords:** Breast, Lymphoma, Non-Hodgkin, Rare Diseases

## Abstract

Non-Hodgkin’s lymphoma of breast is a rare condition. NHL breast constitute about 0.5% of all malignancies of breast. NHL breast constitute nearly 1% of all cases of NHL. Among all subtypes of NHL, DLBCL (Diffuse large B-cell Lymphoma) is the most common type to be known. Marginal zone lymphoma (10-30%), follicular lymphoma (10-20%) and Burkit Lymphoma (5%) are other common histologic variants. Burkitt lymphoma is mainly seen in pregnant females or lactating females. Breast implant associated anapaestic large cell lymphoma (BIA-ALCL) constitutes remaining case. Thus, primary NHL of Breast is rare condition. DLBCL is most common histologic variant. We report here a rare case of primary NHL Breast. A 30 years old lactating mother came with history of swelling and nipple discharge from bilateral breast. -Treatment approach for low grade NHL breast is Radiotherapy only and for high grade NHL breast there is a role for combined modality approach that is chemotherapy followed by Radiotherapy with or without surgical intervention.

## Background

Malignant Lymphoma is a rare lymphoma arising in the breasts in the absence of previously detected lymphoma localisation. Malignant lymphoma is a rare entity whether it is primary or secondary [1]. Breast lymphomas are of various types but the most common is B cell NHL Primary Non-Hodgkin lymphoma breast is a rare entity [2]. and is known to constitute about 0.5% of all cases of breast malignancies. Paucity of lymphoid tissue in breast accounts for its low prevalence [3]. The neoplasm is mainly seen in middle age and older age individual as compared to younger individual and children. Females are most commonly affected than males. The risk factors are nearly same as of common breast cancer. The risk factors are early menarche, late menopause, sedentary lifestyle, nulliparity, obesity, positive family history, genetic mutation etc.

Among all risk factors positive family history is the most common risk factor to be known. Diagnosis of breast lymphoma is challenging task as it has nonspecific imaging features. Diagnosis is made by FNAC or HPE of excised lump [4]. Treatment of primary NHL is a multimodality approach. (Chemotherapy and Radiation therapy), surgery can be avoided [5].

### Case Presentation

A 30 year old female presented in Radiation Oncology OPD in GMCH with chief complaint of swelling in both the breasts for 6 months and bloody discharge from the left nipple for 2 weeks. Patient was apparently well 6 months back when patient noticed swelling in both breasts. The swelling was insidious in onset, mobile and progressive in nature.

Initially swelling was pea sized and now progressed to size of orange. It was associated with pain. The pain was generalised in both the breasts, insidious in onset, sharp, non-radiation to back or shoulder, aggravating on movement and is not relived on medications. The Patient also presented with discharge from left nipple. The discharge was bloody not associated with any blood clots.

There is no significant past history (no history of Tuberculosis/Diabetes/Hypertension/asthma).

The patient does not give any history of malignancy in family.

### On examination Inspection

An ill defined ulcerated mass was present on left breast

Margins of mass were everted

Bleeding from mass was present

Symmetry of both Breasts was distorted

Nearly (6*6) cm mass was seen in left breast

**Figure 1:**
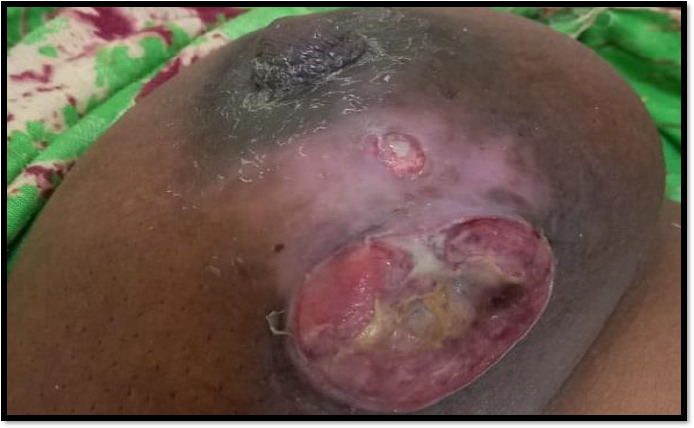
Shows an ulcerated mass with everted edges, with nipple areola complex distorted

Around (5*5) cm mass was palpated in right breast (Figure 1)

### Palpation

A hard, tender, ill defined margin ulcerated mass wass presented in left breast.

Mass was fixed to chest wall

Non-mobile

A hard tender, lump was present in right breast, which was having rubbery constituency, mobile.

### Per-abdomen examination

A hard mass was felt in right hypo gastric region, tender, non-mobile.

### Investigation USG-Abdomen

1. Liver- Borderline hepatomegaly with slightly distorted echo texture.
2. Gall bladder-Distended

### Mammography B/L Breast

1. A well defined lesion in outer quadrant of right breast showing pressure increase in enhancement (type 1) curve and diffusion restructures. (BIRADS-2)
2. Few other tiny well defined lesion in right breast, largest in retroareolar region showing dynamic enhancement with type II curve likely benign lesion (BIRADS-2)

A large lobulated lesion in lower outer quadrant showing heterogeneous signal with central necrotic area well defined margins and progressive increase in enhancement with skin thickening and surrounding inflammation.

**Figure 2 and 3:**
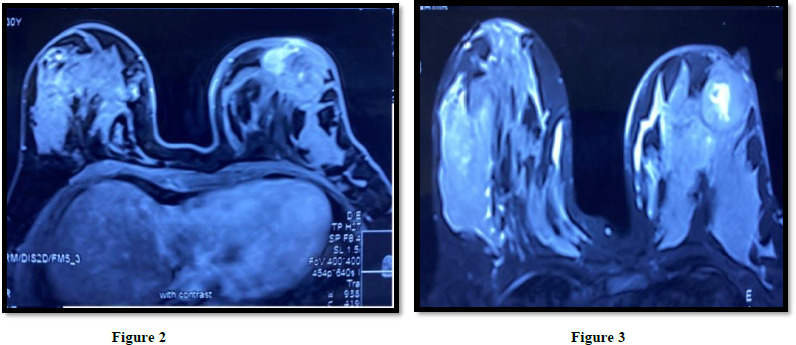
Well defined lesion in both breasts (Mammography B/L Breast)

USG-B/L Breast: Multiple SOL seen in both breast with vascularise (Figure 2 and 3).

### Histopathological examination

Trucut Biopsy-Non-Hodgkin lymphoma (left breast)

Trucut Biopsy-Descriptive (Right breast)

**Figure 4:**
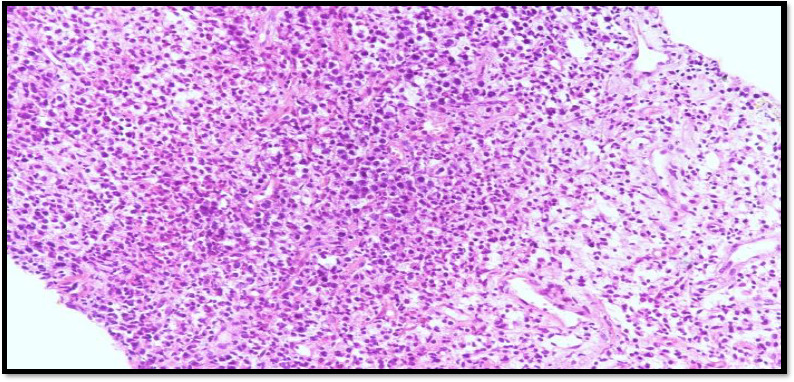
Tumour cell shows hyper chromatic nuclei and moderate amount of cytoplasm

Tumour comparison of solid sheets, discrete small to medium sized round cells. The individual tumour cell shows hyper chromatic nuclei and moderate amount of cytoplasm (Figure 4).

### On IHC:

Tumour cell shows positivity for LCA, CD-3, CD-99

**Figure 5:**
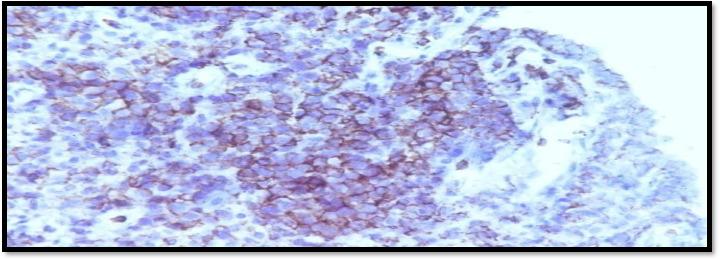
LCA

**Figure 6:**
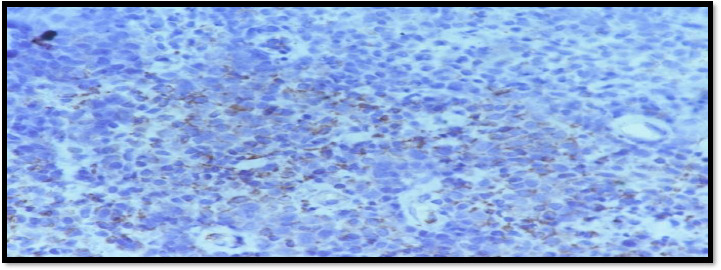
CD-3

**Figure 7:**
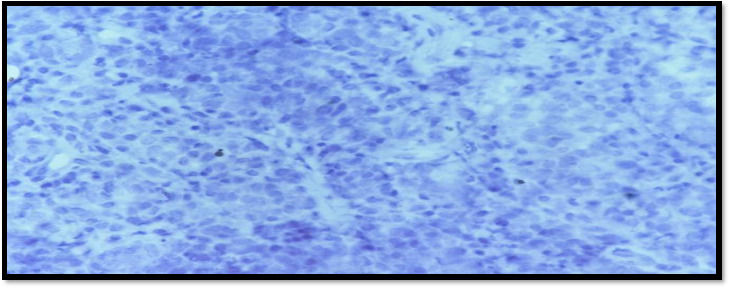
CD-20

Tumour cell show negative for Cytokeratin, CD-20 (Figure 5, 6 and 7)

### Treatment

Treatment approach of primary NHL Breast is based on Radiological investigation, Biopsy and IHC findings. If IHC is positive for CD 20 then, there is role of Rituximab in treatment and if CD 20 is negative there is no role for Rituximab. Treatment approach for low grade NHL breast is Radiotherapy only and for high grade NHL breast there is a role for combined modality that is chemotherapy followed by Radiotherapy with or without surgical intervention. In high grade NHL breast there is also role for prophylactic cranial irradiation as there is high chances of brain metastasis. Thus, Treatment of primary NHL breast is a multi-modality approach.

**Figure 8:**
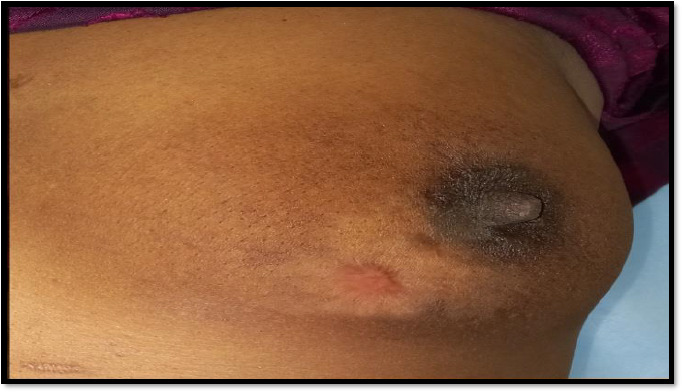
Response after 6 cycles of CHOP regimen chemotherapy

In this case, the tumor was CD20 negative. So, we started the treatment with CHOP regimen (Figure 8).

(Inj. Cyclophosphamide, Inj. Mesna, Inj. Adriamycin, Inj. Vincristine, Tab. Prednisolone)

**Figure 9:**
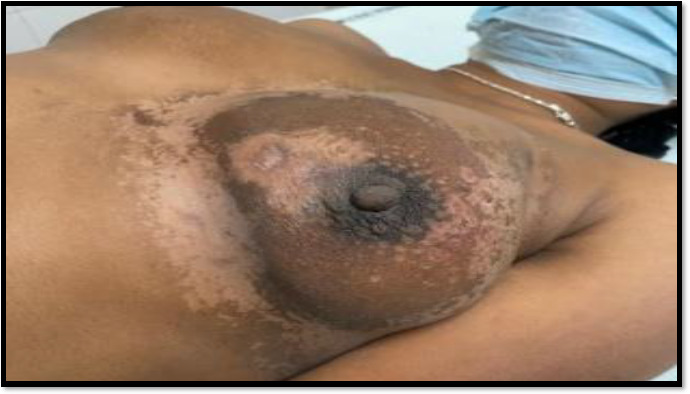
Response after 6 cycles of chemotherapy and radiation to breast

Thus, here our plan was to give 6 cycle of CHOP followed by Radiotherapy to primary disease site and prophylactic cranial irradiation. Now, later depending on the response assessment, we can add or omit surgery. Lesion in the right breast remained as such since it was BIRADS 2 on sonomammography and biopsy was descriptive. The lesion was kept on observation (Figure 9).

## Discussion

Malignant Lymphoma is a rare neoplasm which originates from lymphatic tissue, whereas extranodal Malignant Lymphoma originates in intestine and waldeyer ring [6]

### Incidence

NHL of the breast is not so common [7] accounting for about 1-2 % of all extranodal lymphomas with most common presentation in advanced stage [8].

### Clinical features

It usually manifests as palpable mass (usually in superoexternal quadrant) which may be associated with pain and palpable axillary lymph nodes. Other rare presentations include retraction of skin and nipple discharge, ulceration or “peau d’orange. Features like fever, night sweats, weight of loss are grouped under B symptoms may also be the presenting symptoms rarely [9].

### Diagnosis

Imaging modalities like Mammography, USG and MRI are nonspecific. Biopsy is needed to distinguish NHL mammary gland from classical carcinoma of breast because both present with similar clinical features. IHC is required to classify lymphoma into its subtypes.

### Management

There is no universal treatment protocol for NHL till date [8]. DeCosse JJ, et al. reported that tumours localised to the breast treated with surgical intervention alone had high rates of relapses after 10 years of surgery [10]. In a multicentric study on 84 breast lymphoma patients from 20 institutes showed 95% local control at 5 years with radiation of 40 Gy [11]. Multimodality approach comprising of radiation therapy, chemotherapy and surgery is proven to be effective. CHOP is the most common agent being used in these cases. Since our patient was CD 20 negative, Rituximab was not considered in this case. Adjuvant Radiotherapy minimizes the cardiac toxicity associated with Doxorubicin. Bone marrow transplantation can be considered as an option in refractory or relapsed cases.

## Conclusion

Primary non-Hodgkin lymphoma of breast in 30 years old female is a rare neoplasm. This primary NHL Breast constitutes nearly 0.5 % of all breast malignancies and nearly 1% of all cases of NHL.As, extra nodal involvement of breast is a rare constitute with involvement of lymph node in lymphoma. Primary Non-Hodgkin lymphoma breast have good prognosis. So, aggressive management is carried out to treat the tumour.

Till date treatment approach is controversial. Chemotherapy followed by Radiotherapy is primary treatment. Further treatment will depend on response assessment. So, Further reporting of such case (<0.5%) cases in treatment is of para-amount important to guide the disease risk factor, prognosis and treatment approach.
